# Prediction of Chemical Composition of Alfalfa Blocks Using FTIR Spectroscopy and Machine Learning Models

**DOI:** 10.3390/foods15193375

**Published:** 2026-09-22

**Authors:** Haijun Du, Yanhua Ma, Liying Cao, Hongzhou Liu, He Su, Xiangbo Liu

**Affiliations:** 1 College of Mechanical Engineering, Inner Mongolia University of Science and Technology, Baotou 014010, China; du15563886115@163.com; 2 College of Mechanical and Electrical Engineering, Inner Mongolia Agricultural University, Hohhot 010018, China; liuhongzhou0908@163.com (H.L.); suhe0826@126.com (H.S.); a1546477861@outlook.com (X.L.)

**Keywords:** alfalfa blocks, FTIR, chemical composition, CARS, prediction

## Abstract

Alfalfa blocks are an important commercial roughage, and their crude protein (CP), starch, fat, neutral detergent fibre (NDF), and acid detergent fibre (ADF) contents are key indicators of nutritional quality. This study investigated Fourier-transform infrared (FTIR) spectroscopy combined with machine learning for rapid quantitative prediction of these constituents. Different spectral preprocessing strategies and variable-selection methods were compared using partial least squares regression (PLSR), random forest regression (RFR), and back-propagation (BP) neural networks. The effects of spectral preprocessing and preceding baseline correction varied among constituents, indicating that baseline correction did not uniformly improve predictive performance. Competitive adaptive reweighted sampling (CARS) reduced spectral dimensionality, and the resulting PLSR models outperformed the RFR and BP models for all five constituents in the reported comparisons, with values reported as mean ± standard deviation across 20 repeated data partitions. For CP, the PLSR model combining first-derivative Savitzky–Golay preprocessing with CARS (SG1D-CARS-PLSR) achieved a prediction-set coefficient of determination (R^2^) of 0.961 ± 0.021 and a residual predictive deviation (RPD) of 5.629 ± 1.245. For starch, SG1D-CARS-PLSR achieved an R^2^ of 0.877 ± 0.023 and an RPD of 2.959 ± 0.291. For NDF, the PLSR model combining adaptive iteratively reweighted penalised least-squares baseline correction with CARS (BC-airPLS-CARS-PLSR) achieved an R^2^ of 0.986 ± 0.006 and an RPD of 9.355 ± 1.947. For ADF, BC-airPLS-CARS-PLSR achieved an R^2^ of 0.839 ± 0.031 and an RPD of 2.591 ± 0.287. For fat, BC-MSC-CARS-PLSR achieved an R^2^ of 0.697 ± 0.058 and an RPD of 2.1.883 ± 0.164, and did not deliver the expected stable prediction performance as predefined. These findings highlight the importance of constituent-specific preprocessing and variable selection and demonstrate that the optimised FTIR-based models provide a rapid for estimating the five constituents from a single spectrum, with an analysis time considerably shorter than that of the reference wet-chemistry methods.

## 1. Introduction

In recent years, Fourier-transform infrared spectroscopy (FTIR) has been widely applied in the fields of quality monitoring and compositional analysis of agricultural products, owing to its rapidity, reproducibility, and operational simplicity. When combined with chemometrics and machine learning approaches, FTIR enables the rapid quantitative prediction of the chemical constituents of forage and its processed products, thereby providing an efficient technical means for the quality evaluation of forage [1,2]. Owing to its high protein content, strong adaptability, and rich nutritional value, alfalfa is one of the most important high-quality leguminous forages in modern grassland agriculture and ruminant production [3].

Alfalfa blocks are feed products manufactured by compressing and moulding alfalfa meal. They offer advantages in storage and transportation, loss reduction, and improved utilisation efficiency. However, variations in raw material source, grinding degree, moisture content, and compression-processing conditions may lead to substantial differences in the physical structure of alfalfa blocks and the state of the internally contained alfalfa meal, consequently affecting their nutritional composition and feeding value [4,5]. Crude protein (CP), starch, fat, neutral detergent fibre (NDF), and acid detergent fibre (ADF) are key parameters for evaluating the nutritional quality and feeding value of alfalfa-based feed products [6]. Accurate and timely determination of these constituents is essential for quality grading of alfalfa blocks, optimisation of feed formulations, and efficient livestock production.

Although traditional chemical analysis methods possess relatively high accuracy, they are generally associated with complex sample preparation, lengthy detection times, substantial reagent consumption, and high analytical costs, which renders them inadequate for meeting the rapid detection demands of large numbers of forage samples in large-scale livestock production. With the advancement of spectral detection technology and data-driven modelling approaches, rapid quality-detection techniques based on infrared spectroscopy have provided a feasible solution to the aforementioned problems [3]. The positions, intensities, and shapes of absorption peaks in infrared spectra are closely related to the vibrational modes of chemical bonds and the characteristics of functional groups within the sample [7,8]; consequently, infrared spectroscopy can be employed to characterise the content variations in components such as proteins, lipids, starch, and fibre [1,9]. Although conventional chemical analyses provide reliable reference measurements, they are time-consuming, reagent-intensive, and less suitable for high-throughput forage screening. Infrared spectroscopy therefore offers an attractive alternative for rapid compositional analysis. Near-infrared spectroscopy (NIRS) has been successfully applied to the prediction of protein and other chemical constituents in agricultural materials, including seaweed and rice [8,10]. In comparison, FTIR provides more distinct fundamental vibrational bands associated with specific functional groups, which can facilitate chemical interpretation. FTIR has also been successfully used for rapid crude-protein determination in alfalfa [11]. direct transfer of such models to compressed alfalfa blocks cannot be assumed. Alfalfa-block production involves densification under different moisture levels, die geometries, and compression conditions, which can induce thermomechanical and microstructural changes in the material. Some of these processing-induced changes may persist even after subsequent grinding and may influence particle organization, matrix interactions, scattering behaviour, and the resulting infrared spectral response. Therefore, the present study extends the previous work on loose alfalfa meal by focusing on processed alfalfa blocks produced under different densification conditions and by evaluating multiple spectral preprocessing, variable-selection, and regression strategies for the simultaneous prediction of CP, starch, fat, NDF, and ADF. This distinction represents the principal methodological and application-oriented advance over the earlier study by Du et al. [11]. Therefore, the present study focused on FTIR combined with spectral preprocessing, CARS variable selection, and machine-learning regression for the prediction of CP, starch, fat, NDF, and ADF in alfalfa blocks.

The performance of spectral prediction models depends largely on data preprocessing, feature-variable selection, and regression-modelling strategies. Spectral preprocessing can reduce interference arising from instrumental noise, scattering effects, baseline drift, and environmental factors. For example, Savitzky–Golay (SG) smoothing effectively suppresses high-frequency random noise, whereas derivative transformations facilitate the separation of overlapping absorption peaks and correction of baseline drift [7]. Subsequently, feature extraction can identify informative wavenumber variables closely associated with the target constituents, thereby reducing redundant information and data dimensionality and improving model computational efficiency, robustness, and prediction accuracy [12]. Competitive adaptive reweighted sampling (CARS), successive projections algorithm (SPA), and principal component analysis (PCA) have been widely used in spectral modelling of agricultural products [13,14]. In particular, CARS exhibits strong variable-selection capability for high-dimensional spectral datasets with pronounced collinearity [15].

Regression modelling is another essential component of constituent-content prediction, and different model architectures may exhibit substantial differences in fitting capacity and generalisation performance when processing spectral data. Common modelling approaches include partial least squares regression (PLSR), random forest regression (RFR), and back-propagation (BP) neural networks [16]. For example, Shi et al. combined near-infrared spectroscopy with PLSR to develop a model for rice samples. Following second-derivative preprocessing and variable selection using CARS and Monte Carlo uninformative variable elimination (MC-UVE), the coefficient of determination for the prediction set (R^2^) was improved to 0.97, enabling rapid and accurate determination of rice protein content [10]. BP-based quantitative models have also demonstrated excellent nonlinear fitting capability, high detection accuracy, and low prediction errors, with reported values of R^2^ > 0.98 and root mean square errors (RMSEs) below 0.04 [16].

Although numerous studies have reported the prediction of chemical constituents in loose alfalfa meal using spectroscopic techniques, FTIR-based detection and compositional prediction for compressed alfalfa blocks remain relatively limited. The dense structure and internal particle-state characteristics of alfalfa blocks may affect their spectral responses, thereby limiting the direct transferability of models developed for alfalfa meal samples. Therefore, this study collected FTIR spectra from alfalfa blocks produced using different densification processes and combined different spectral preprocessing methods, feature-extraction algorithms, and machine learning-based regression models to establish prediction models for key chemical constituents, including CP, starch, fat, NDF, and ADF. The predictive performance of different modelling combinations was systematically compared. The results are expected to provide technical support for the rapid and accurate evaluation of the nutritional quality of alfalfa blocks.

## 2. Materials and Methods

### 2.1. Materials

Alfalfa samples were obtained from the experimental base of Inner Mongolia Agricultural University and the agro-pastoral ecotone experimental base of the Grassland Research Institute, Chinese Academy of Agricultural Sciences. The material consisted of Zhongcao No. 13 alfalfa. A total of 137 alfalfa-block samples produced using different densification processes were randomly collected. The samples were dried in an oven at 60 °C until a constant weight was achieved and were subsequently ground into fine powder using a grinder.

The alfalfa raw materials for the 137 samples were obtained from the experimental field of Inner Mongolia Agricultural University and the alfalfa planting base of the Grassland Research Institute, Chinese Academy of Agricultural Sciences; materials from both sources were used for the densification tests. Single-factor tests were conducted: the moisture content was adjusted from 14% to 20%, the die length-to-diameter ratio from 4.7 to 5.9, and the compression speed was set at 3, 5, 7, and 9 mm·s^−1^, while the remaining processing parameters were kept unchanged as specified above. In addition, a full-factorial experimental design was adopted, with three moisture content levels (15.0%, 16.5%, and 18.0%), three die length-to-diameter ratios (5.0, 5.3, and 5.6), and three compression speeds (5, 7, and 9 mm·s^−1^).

Alfalfa protein content was determined according to the Chinese national standard Determination of Crude Protein in Feed—Kjeldahl Method [17]; alfalfa fat content was determined according to the Chinese national standard Determination of Crude Fat in Feed [18]; alfalfa neutral detergent fibre (NDF) content was determined according to the Chinese national standard Determination of Neutral Detergent Fibre in Feed [19]; alfalfa acid detergent fibre (ADF) content was determined according to the Chinese agricultural industry standard Determination of Acid Detergent Fibre in Feed [20]; and alfalfa starch content was determined according to the Chinese national standard Determination of Starch in Foods [21]. Each sample was analysed in triplicate, and the mean of the three replicates was used as the reference value for calibration. For the sample showing the greatest variability among replicates, the sample standard deviation (SD, n − 1) and coefficient of variation (CV) were 0.075 and 0.4% for crude protein, 0.21 and 1.6% for starch, 0.153 and 4.7% for fat, 0.737 and 1.8% for NDF, and 1.677 and 5.0% for ADF, respectively. These values represent the upper bound of within-sample variability of the reference analyses. Averaging the triplicates reduces the random error of the reference values by a factor of $\sqrt{3}$(SE = SD/$\sqrt{3}$), thereby limiting the transfer of reference-method error into the calibration models.

### 2.2. FTIR Acquisition

An FTIR-1500 Fourier transform infrared spectrometer (Zhengzhou Aoda Instrument Co., Ltd., Zhengzhou, China) was used to acquire spectral data from the alfalfa-block samples. Spectra were collected over the range of 4000–400 cm^−1^, with a resolution of 4 cm^−1^ and 16 scans per measurement. The instrument was warmed up for 0.5 h before sample scanning.

For each sample, 0.02 g of dried alfalfa meal and 2.5 g of potassium bromide (KBr) were randomly weighed and ground in a quartz mortar with a pestle until a homogeneous, fine powder was obtained. The mixture was then pressed into a test pellet using a hydraulic press and placed in the spectrometer for spectral acquisition. Raw spectral data were collected using the instrument’s built-in FTIR software V1.2.0.1. Before acquiring the spectrum of each sample, an air-background spectrum was recorded to minimise interference from environmental factors. Each sample was measured ten times at randomly selected positions on the same KBr pellet, and the mean spectrum was used to construct the spectral profile for subsequent analysis. Because all ten spectra were recorded from a single pellet, the replicate variability characterises instrumental repeatability and local pellet inhomogeneity rather than pellet-to-pellet preparation variability. The spectrometer available to this study (FTIR-1500) was equipped with a transmission accessory only. Excluding oven drying, which is common to the reference methods, grinding, pellet preparation and ten spectral acquisitions required 13–15 min per sample.

### 2.3. Spectral Preprocessing Methods

The raw spectra showed broad baseline offsets and drift, potentially arising from KBr-pellet thickness variation, particle scattering, residual moisture in KBr, and instrumental background variation. Therefore, two parallel analysis branches were evaluated. In the first branch, raw spectra were directly subjected to subsequent spectral preprocessing. In the second branch, spectra were first processed using the optional baseline-correction (BC) function provided with the FTIR instrument software V1.2.0.1 and were subsequently subjected to spectral preprocessing. Specifically, BC was implemented through the data-preprocessing module provided with the FTIR instrument software, using a third-order polynomial baseline-fitting setting to correct broad baseline offsets and low-order drift in the spectral data, as illustrated in Figure 1.

Savitzky–Golay (SG) smoothing was applied with a 15-point window and a third-order polynomial. The first derivative (SG1D) was then computed from the smoothed spectra with a 15-point window and a first-order polynomial. The effect of each pretreatment was assessed by 5-fold cross-validated PLS regression on the full spectra. In addition, multiplicative scatter correction (MSC) and adaptive iteratively reweighted penalised least squares (airPLS) were applied.

In addition, since airPLS itself can perform both baseline estimation and baseline removal, the algorithm was tested both on the raw spectra and, as an exploratory sequential analysis condition, on the spectra processed with BC (BC + airPLS).

### 2.4. Feature Extraction Methods

Feature selection was performed to address the large number of spectral variables and to eliminate wavelength features unrelated to the measured chemical components. The aim was to identify the most relevant and informative variables, thereby improving model accuracy, reducing computational cost, and enhancing model interpretability. PCA, SPA, and CARS were employed for feature selection. PCA projects high-dimensional and highly correlated spectral bands into a small number of mutually uncorrelated principal components. This approach retains the maximum spectral variance while reducing dimensionality, redundancy, and noise, thereby improving the efficiency of subsequent classification or quantitative modelling [22]. Spectra were standardised before decomposition. The number of components was the smallest giving ≥ 95% cumulative explained variance; their scores were used as regressors.

SPA sequentially selects spectral bands with the largest projection-vector angles, yielding feature variables with low collinearity and highly complementary information. Thus, SPA reduces spectral redundancy using fewer wavelengths and improves model robustness and interpretability [23]. SPA was applied to standardised spectra. The first variable was that of maximum variance; each subsequent variable maximised the projection norm onto the orthogonal complement of the selected set. Chains of up to 300 variables were built; for each length a multiple linear regression was fitted on the training samples and evaluated on the validation samples, and the length with minimum validation RMSE was retained.

CARS adaptively selects an optimal subset from a large number of variables, substantially reducing model complexity while improving prediction accuracy. These methods evaluate the relationship between each wavelength and the target variable by considering factors such as the signal-to-noise ratio, spectral characteristics, and associations with underlying physical or chemical processes [10]. Fifty Monte Carlo runs were performed. In each run 80% of the calibration samples were drawn without replacement, a PLS sub-model with [n] LVs was fitted and variables were ranked by the absolute normalised regression coefficients. The retained fraction in run i followed r_i_ = 0.9·exp(−i/50). The subset with the lowest 5-fold RMSECV over the 50 runs was selected. Ensure that all data from the test set are retained in the PLSR test set.

### 2.5. Prediction Models

This study aimed to develop rapid FTIR-based prediction models for the simultaneous estimation of five nutritional constituents (CP, starch, fat, NDF, and ADF) in alfalfa blocks, where “rapid” refers to the analysis time relative to the reference wet-chemistry methods (about 13–15 min per sample excluding oven drying; Section 2.2). PLSR, RFR, and BP neural networks were employed.

PLSR is a classical linear-regression method widely used in spectral analysis. It effectively addresses multicollinearity by simultaneously projecting predictor and response variables into a new latent-variable space, thereby identifying relationships between them. Before modelling, the spectral predictor matrix was autoscaled using a StandardScaler, i.e., each wavenumber variable was mean-centred and scaled to unit standard deviation; the scaling parameters (mean and standard deviation) were estimated exclusively from the training data of each cross-validation fold or from the combined training–validation data used to fit the final model, and then applied to the corresponding validation/test data, so as to prevent information leakage from the validation or test sets into the scaling step. The response variable was not externally standardised. The internal scaling option of PLSRegression was set to scale = False to avoid duplicate (re-)scaling of the already-standardised predictor variables. For each chemical constituent and spectral preprocessing condition, the optimal number of latent variables (LVs) was determined separately. Candidate PLSR models with 1–15 LVs were assessed using shuffled five-fold cross-validation performed on the combined training and validation datasets; the LV number corresponding to the minimum mean cross-validation MSE was selected. The final PLSR model was then refitted using the combined training and validation data with the selected LV number, whereas the independent test set was withheld entirely from training and LV selection and used exclusively for final, unbiased performance evaluation. This entire procedure (data splitting, LV selection, model fitting and evaluation) was repeated for 20 independent random partitions to quantify the variability of the reported performance metrics.

RFR constructs multiple decision trees and integrates their predictions, enabling automatic capture of complex interactions among spectral features without explicitly specifying variable relationships. It is also robust to outliers and noise and can identify spectral regions contributing most to prediction through its built-in feature-importance evaluation mechanism [24]. Hyperparameters were selected by grid search over number of trees (100, 200, 300), features per split (all, √p, log_2_p), maximum depth (10, 20, unlimited), minimum samples to split (2, 5, 10) and minimum samples per leaf (1, 2, 4), 243 combinations in total, using validation-set MSE; the final model was refitted on training + validation data (seed 52).

BP neural networks iteratively adjust network weights through back-propagation to learn nonlinear relationships between spectral bands and target properties, automatically extract key features, and improve prediction accuracy for complex spectral modelling [15]. The network consisted of an input layer containing the full spectral variables, one hidden layer with 12 neurons, and one output neuron for quantitative prediction. The hyperbolic tangent activation function was used in the hidden layer. Model parameters were optimised using back-propagation with the Adam optimiser, with an initial learning rate of 0.001 and an L2 regularisation coefficient of 0.001. The maximum number of training epochs was set to 5000. Early stopping was applied to reduce overfitting; 15% of the training data was internally reserved for monitoring validation performance. Training was stopped when the validation score failed to improve by at least 1 × 10^−6^ for 150 consecutive epochs, or when the maximum number of epochs was reached. Prior to model development, the spectral predictors and response values were independently normalised to [−1, 1] using Min-Max scaling. The scaling parameters were fitted exclusively on the training set and then applied to the validation and independent prediction sets. RMSE was calculated on the original response scale for BPNN. For BPNN, predictions were first inverse-transformed from the normalized [−1, 1] range to the original scale. Thus, RMSE has the same unit as the corresponding reference measurement; for constituents expressed as percentages, RMSE is reported in percentage points.

For the final comparison of regression algorithms, PLSR, RFR, and BP models were evaluated using the same 20 repeated random data partitions. For each partition, the dataset was divided into training, validation, and test sets using the predefined proportions, and the same sample assignments were used for all three model types to ensure direct comparability. The reported model-comparison metrics represent the mean values across the 20 repeated partitions.

### 2.6. Model Evaluation Metrics

Regression-model performance was evaluated using the coefficient of determination for the test set (R^2^), root mean square error (RMSE), and residual predictive deviation (RPD). The calculation formulas for these evaluation metrics are given in Equations (1)–(3). RMSE is defined as the square root of the mean squared difference between predicted and reference values:

(1) $$RMSE=\sqrt{\frac{1}{n}\sum_{i=1}^{n}{\left(y_{i}-{\hat{y}}_{i}\right)}^{2}},$$

where n is the number of samples, $y_{i}$ is the reference value of the i-th sample, and ${\hat{y}}_{i}$ is its predicted value. RMSE was calculated as the square root of the mean squared difference between the measured and predicted values. Since the response variables in PLSR were not standardized, RMSE was expressed in the same units as the corresponding reference measurements; for constituents expressed as percentages, RMSE is reported in percentage points.

The coefficient of determination measures the proportion of variation in the dependent variable explained by the model:

(2) $$R^{2}=1-\frac{\sum_{i=1}^{n}{\left(y_{i}-{\hat{y}}_{i}\right)}^{2}}{\sum_{i=1}^{n}{\left(y_{i}-{\bar{y}}_{i}\right)}^{2}},$$

where ${\bar{y}}_{i}$ is the mean of the reference values. It represents the proportion of data variance explained by the model.

The residual predictive deviation (RPD) is defined as the ratio of the standard deviation of the reference data to the standard error of prediction:

(3) $$\mathrm{RPD}=\frac{\sqrt{\frac{1}{n-1}\sum_{i=1}^{n}{\left(y_{i}-{\bar{y}}_{i}\right)}^{2}}}{\sqrt{\frac{1}{n}\sum_{i=1}^{n}{\left(y_{i}-{\hat{y}}_{i}\right)}^{2}}},$$

The RPD values were interpreted as follows: RPD < 1.5 indicates an unusable model; 1.5 < RPD < 2.0 indicates classification purposes; 2.0 < RPD < 2.5 indicates approximate prediction; 2.5 < RPD < 3.0 indicates good potential for prediction; and RPD > 3.0 indicates excellent prediction suitable for precise quantitative applications [25].

### 2.7. Experimental Setup and Configuration

When performing FTIR analysis on alfalfa pellets, 137 samples were divided into training, validation, and prediction sets in a 7:1.5:1.5 ratio. The division was done using stratified sampling based on the quantile bins of the target variable y (make_strata function, with the default of N_STRATA = 5 layers). Spectral preprocessing, feature extraction (PCA, SPA, and CARS), and model construction were implemented in Python 3.8: PLSR, RFR, and PCA were built on scikit-learn; SPA and CARS were implemented with in-house Python 3.8 scripts; and the BP neural network was implemented with the PyTorch 2026.1.4 framework. All computations were performed on a desktop computer equipped with an AMD Ryzen 9 7950X CPU with 64 GB RAM running on Windows 11, without GPU acceleration. The versions of the main Python packages used were numpy 1.24.4, scipy 1.10.1, and scikit-learn 1.3.2.

## 3. Results and Discussion

### 3.1. Spectral Analysis

The raw FTIR spectra of alfalfa blocks exhibited typical Fourier-transform infrared spectral characteristics (Figure 2). The absorption peaks primarily originated from molecular vibrations, including stretching and bending modes. Within the spectral range of approximately 4000–400 cm^−1^, several distinct absorption peaks were observed, mainly attributed to O–H, N–H, and C–H bond vibrations [26,27]. The absorption bands in the 3000–2800 cm^−1^ region were mainly attributed to aliphatic C–H stretching vibrations of CH_2_ and CH_3_ groups. Protein-associated bands include amide I near 1650 cm^−1^, arising predominantly from peptide C=O stretching, and amide II near 1540 cm^−1^, arising mainly from coupled N–H bending and C–N stretching vibrations. The amide I region may also overlap with water bending absorption near 1640 cm^−1^ [10]. Lipid-related absorption bands are primarily found in two regions. In the high-wavenumber region, they include C-H stretching vibrations: symmetric stretching of CH2 and CH3 at 2880–2850 cm^−1^, and asymmetric stretching at 2970–2950 and 2930–2920 cm^−1^ [28,29]. In the lower-wavenumber region, lipid bands include C=O stretching vibrations at 1720–1750 cm^−1^ and C-O-C stretching vibrations at 1070–1250 cm^−1^ [30,31]. The stretching vibrations of C-O-C and C-O-H in starch occur near 939 cm^−1^. The spectral region of 1050–1245 cm^−1^ is associated with α-1,4-glycosidic bonds (C-O-C) and glucose units (C-C) [3,32]. Characteristic bands of fibre-related O-H and C-H functional groups occur in the 1650–1500 cm^−1^ region, while the O-H stretching band of water and cellulose is observed at 3340–3250 cm^−1^ [33,34]. Detailed analysis of the absorbance, bandwidth, and positions of these bands provides a chemical basis for the prediction of major nutritional constituents in alfalfa blocks, including proteins, lipids, carbohydrates, and structural fibre components. However, because FTIR bands are broad and frequently overlap, individual wavenumbers cannot always be assigned uniquely to a single constituent. Therefore, CARS was subsequently used to identify the subset of spectral variables that contributed most strongly to the prediction of each nutritional parameter.

### 3.2. Nutritional Data Analysis of Alfalfa Blocks

The contents of CP, NDF, ADF, fat, and starch were evaluated in 137 alfalfa-block samples. The results showed a significant correlation between NDF and ADF (Figure 3). CP ranged from 16.69% to 20.73%, fat from 3.2% to 4.5%, starch from 11.6% to 15.6%, ADF from 30.65% to 36.56%, and NDF from 39.39% to 45.11%. Except for NDF and ADF, all other nutrients exhibited coefficients of variation (CVs) greater than 5%, indicating considerable variability among the samples. The broad ranges of nutrient contents demonstrate good sample representativeness. Significant differences in nutrient concentrations among samples are beneficial for developing representative and robust FTIR prediction models.

### 3.3. Results of Spectral Preprocessing

As shown in Figure 4, the raw FTIR spectra were preprocessed using SG smoothing, SG1D, MSC, and airPLS. Without baseline correction (Figure 4a), the original spectra exhibited evident baseline drift and intensity offsets across the full spectral range, particularly in the low-wavenumber region, which may mask weak absorption features. SG smoothing effectively reduced high-frequency random noise while preserving the overall shape of the main absorption bands. SG1D further enhanced spectral resolution, making adjacent or overlapping absorption features more distinguishable; however, it also amplified local noise [35]. MSC reduced multiplicative and additive scattering effects caused by particle scattering, sample thickness, and optical path-length differences, thereby improving spectral comparability among samples. AirPLS adaptively estimated and removed the background baseline, effectively correcting nonlinear baseline shifts associated with instrumental drift, scattering, and other background effects [36].

After airPLS baseline correction (Figure 4b), overall baseline drift was substantially suppressed, and absorption features became more concentrated around the zero baseline while retaining the positions and relative trends of the main characteristic peaks. Compared with the uncorrected spectra, the SG, SG1D, and MSC treated spectra after baseline correction showed more stable baselines and more consistent spectral features.

Furthermore, PLSR models were used to evaluate the effects of different preprocessing methods on the prediction of CP, fat, starch, NDF, and ADF, as summarised in Table 1. Model performance was primarily assessed using the R^2^ and residual predictive deviation (RPD) values for the calibration and test sets. Generally, higher R^2^ and RPD values indicate better model fit and more stable predictive performance. The optimal LV number for each component–preprocessing combination was selected via 5-fold cross-validation on the combined training and validation data (the LV number minimising the mean CV-MSE), based on a single representative data partition.

For CP prediction, SG1D preprocessing without baseline correction showed the best performance, with R^2^ values of 0.9980 and 0.9621 and RPD values of 22.3110 and 5.2627 for the calibration and test sets, respectively, indicating high prediction accuracy. After baseline correction, the performance of the SG1D model declined markedly. In comparison, airPLS provided relatively better test-set results (R^2^ = 0.4609, RPD = 1.3955), although its overall predictive ability remained limited.

For fat, the SG1D model performed relatively well before baseline correction, achieving a test-set R^2^ of 0.5235 and an RPD of 1.4844. After baseline correction, MSC achieved the best prediction performance, with calibration-set R^2^ and RPD values of 0.8696 and 2.7841, respectively, and test-set values of 0.5486 and 1.5252. These results suggest that MSC can partially mitigate the effects of spectral scattering on fat prediction; however, further improvement in test-set predictive performance is still needed.

For starch, SG1D preprocessing without baseline correction produced the best prediction performance, with a test-set R^2^ of 0.8898 and an RPD of 3.0867, indicating good quantitative predictive capability. Following baseline correction, the test performance of the SG1D model decreased (R^2^ = 0.8365, RPD = 2.5343), but it remained superior to the other preprocessing methods. AirPLS combined with baseline correction also provided good results, with a test-set R^2^ of 0.7940 and an RPD of 2.2579.

For NDF and ADF, the different preprocessing methods achieved high fitting performance in the calibration sets; however, their test-set results were generally unstable, with some models even showing negative R^2^ values. This indicates insufficient generalisability and possible overfitting. For NDF, airPLS after baseline correction provided relatively better test-set performance (R^2^ = 0.6278, RPD = 1.6796). Similarly, for ADF, airPLS after baseline correction showed the best relative performance (R^2^ = 0.5710, RPD = 1.5644). Nevertheless, the RPD values for both traits were below 2, suggesting that the current models are more suitable for preliminary screening or trend analysis than for high-precision quantitative prediction.

In summary, preprocessing methods significantly affected the performance of PLSR models for different nutritional components, and the optimal method varied by component. SG1D was more suitable for predicting CP and starch, with the starch model showing good potential for practical application. MSC offered certain advantages for fat prediction, whereas airPLS baseline correction was relatively beneficial for developing NDF and ADF models. Overall, baseline correction did not consistently improve predictive performance for all components. Therefore, preprocessing strategies should be selected according to the characteristics of the target component and the performance on an independent test set [37].

### 3.4. Feature Extraction Results

After determining the optimal preprocessing strategy for each component, PCA, SPA, and CARS were further employed for feature-variable extraction to reduce the interference of redundant variables and noise in full-spectrum modelling. Based on the PLSR model mentioned in Section 2.5 and the parameter settings, a comparative selection of feature extraction algorithms is performed. As shown in Table 2, PLSR models developed using CARS-selected variables generally achieved the best or near-best predictive performance for all components. This indicates that CARS can effectively retain spectral variables closely associated with the target component concentrations while eliminating uninformative and collinear variables. In contrast, PCA is an unsupervised dimensionality-reduction method, and the extracted principal components are not necessarily directly related to the concentrations of the target components. Although SPA can reduce variable collinearity, the selected variables may provide insufficient coverage of overall spectral information for certain components, thereby limiting model generalisability.

In summary, feature-variable extraction significantly influenced the predictive performance of FTIR–PLSR models. CARS achieved the highest test-set R^2^ and RPD values, together with the lowest RMSE values, for CP, starch, fat, NDF, and ADF prediction, demonstrating superior variable-selection capability and model generalisability. Ultimately, SG1D-CARS was suitable for quantitative prediction of CP and starch; BC-MSC-CARS was suitable for fat prediction; and BC-airPLS-CARS effectively improved the prediction accuracy for NDF and ADF. These results demonstrate that combining component-specific preprocessing with CARS feature selection can substantially enhance the rapid quantitative analysis of nutritional components using FTIR spectroscopy [38].

To reduce the dimensionality of the FTIR spectra and remove redundant spectral information, competitive adaptive reweighted sampling (CARS) was applied separately to the prediction of CP, starch, fat, NDF, and ADF. CARS retained 865, 282, 424, 192, and 424 variables for CP, starch, fat, NDF, and ADF, respectively, from the original 10,329 spectral variables, demonstrating a substantial reduction in input dimensionality. The selected variables and their positions relative to the average FTIR spectrum are shown in Figure 2.

Importantly, the CARS-selected variables were not distributed exclusively in spectrally featureless regions, but showed correspondence with several chemically meaningful absorption regions identified in Section 3.1. For CP, selected variables occurred in or near the protein-associated amide I and amide II regions, around 1650 and 1540 cm^−1^, respectively, supporting the contribution of peptide C=O stretching and coupled N–H bending/C–N stretching vibrations to CP prediction. Variables were also retained in the broad O–H/N–H stretching region at approximately 3340–3250 cm^−1^, which may contain additional information associated with protein and moisture-related hydrogen bonding.

For starch, several selected variables were located in the fingerprint region of approximately 1050–1245 cm^−1^, which is associated with C–O–C vibrations of α-1,4-glycosidic bonds and C–C vibrations of glucose units. This correspondence supports the chemical relevance of the CARS-based variable selection for starch prediction. Selected wavelengths in other regions may reflect indirect compositional correlations or overlapping vibrational contributions among constituents rather than isolated absorption of starch alone.

It should be noted that the spectral assignment in the 1050–1250 cm^−1^ region is not unique to a single chemical constituent. The lipid-related C–O–C band reported at approximately 1070–1250 cm^−1^ substantially overlaps with the starch-associated region at approximately 1050–1245 cm^−1^. Therefore, variables selected within this interval cannot be interpreted as exclusively representing either lipid or starch. Instead, this region contains overlapping vibrational information from multiple C–O and C–O–C containing structures. The different CARS selection patterns obtained for fat and starch indicate that the predictive models exploit combinations of spectral variables across multiple regions rather than relying on a single diagnostic band.

### 3.5. Prediction of Nutritional Components in Alfalfa Blocks Using FTIR

Figure 5 compares the predictive performance of PLSR, RFR, and BP models for CP, starch, fat, NDF, and ADF on the independent test set. Overall, PLSR showed the strongest performance for CP, starch, NDF, and ADF, whereas the three models exhibited more similar performance for fat. For comparisons within the same constituent, RMSE values were calculated on the original response scale and therefore have the same units as the corresponding reference measurements.

For CP, PLSR achieved the highest predictive accuracy, with an R^2^ of 0.961, an RMSE of 0.169, and an RPD of 5.629. By comparison, RFR and BP yielded lower R^2^ values of 0.735 and 0.834, respectively, together with higher RMSE values of 0.513 and 0.355 and lower RPD values of 1.992 and 2.517. These results indicate that the latent-variable representation employed by PLSR was particularly effective for modelling CP-related spectral information.

For starch, PLSR also provided the strongest overall performance, with an R2R^2 of 0.877, an RMSE of 0.308, and an RPD of 2.959. The BP model showed comparable but slightly lower predictive capability (R^2^ = 0.826, RMSE = 0.340, RPD = 2.459), whereas RFR performed less favorably (R^2^ = 0.689, RMSE = 0.592, RPD = 1.638). The relatively close performance of PLSR and BP suggests that both linear latent-variable modelling and nonlinear regression were able to capture starch-related spectral information, although PLSR retained a moderate advantage under the present experimental conditions.

For fat, PLSR and RFR showed relatively comparable predictive performance, whereas BP performed substantially less favorably. PLSR achieved an R^2^ of 0.697 and an RPD of 1.883, compared with 0.664 and 1.767, respectively, for RFR. These results indicate that PLSR provided the strongest overall performance for fat when considering the three evaluation metrics jointly, although the difference between PLSR and RFR was relatively small. The comparatively modest predictive performance for fat may be associated with weaker or more overlapping lipid-related spectral information and the relatively limited variation in the reference values.

The most pronounced advantage of PLSR was observed for NDF. The PLSR model achieved an R^2^ of 0.986, an RMSE of 0.140, and an RPD of 9.355, substantially outperforming RFR (R^2^ = 0.528, RMSE = 0.944, RPD = 1.492) and BP (R^2^ = 0.556, RMSE = 0.743, RPD = 1.539). This result suggests that the spectral variation relevant to NDF was efficiently represented by a low-dimensional latent-variable structure after preprocessing and CARS-based variable selection.

For ADF, PLSR again demonstrated the best overall predictive performance, with an R2R^2 of 0.839, an RMSE of 0.579, and an RPD of 2.591. BP achieved similar performance (R^2^ = 0.812, RMSE = 0.583, RPD = 2.361), whereas RFR showed substantially lower predictive accuracy (R^2^ = 0.561, RMSE = 1.032, RPD = 1.547). The relatively small difference between PLSR and BP indicates that both linear and nonlinear modelling strategies were capable of extracting useful ADF-related information from the selected spectral variables, although PLSR remained slightly more robust.

Taken together, PLSR exhibited the most consistent overall predictive performance across the five nutritional constituents evaluated. Its advantage was particularly pronounced for CP and NDF, for which substantially higher R^2^ and RPD values and lower RMSE values were obtained than with RFR and BP. For starch and ADF, BP provided relatively competitive results, although PLSR retained a modest overall advantage. For fat, PLSR and RFR produced broadly comparable results, with PLSR yielding higher R^2^ and RPD values and RFR showing a slightly lower RMSE. These findings therefore suggest that, under the present sample size, preprocessing strategies, and CARS-selected variable subsets, PLSR provided the most consistent balance of predictive accuracy among the three modelling approaches.

The comparatively weaker performance of RFR and BP for several constituents may partly reflect the limited sample size available for model development. PLSR projects highly collinear spectral variables onto a reduced set of latent variables, thereby reducing the effective dimensionality of the regression problem. Such a structure can be advantageous under small-sample conditions by limiting model complexity and reducing the risk of overfitting. Nevertheless, the stronger performance of PLSR in the present dataset should not be interpreted as evidence that the relationships between FTIR spectra and chemical composition are inherently linear. Larger and more heterogeneous datasets, preferably including samples from independent production batches and sources, would be required to determine whether more flexible nonlinear models can provide additional predictive gains.

In addition, attenuated total reflectance Fourier transform infrared (ATR–FTIR) spectroscopy is a potential alternative to the KBr-pellet transmission method used here. ATR permits direct measurement of prepared powders placed in contact with an ATR crystal, without KBr dilution or pellet pressing. It could therefore simplify sample preparation, shorten preparation time, and avoid variability associated with KBr mixing, pellet thickness, and moisture uptake by the hygroscopic KBr matrix. However, reproducible ATR measurements require adequate and consistent sample–crystal contact, and powder heterogeneity, particle size, and applied contact pressure may affect the spectra. Furthermore, ATR and transmission measurements differ in effective sampling depth and relative band intensities; the present transmission-based calibration models therefore cannot be assumed to apply directly to ATR spectra. ATR was not evaluated because the available instrument was equipped only for transmission measurements. Its potential benefits for alfalfa block analysis should be assessed through a dedicated comparison, including model recalibration and independent validation.

### 3.6. Discussion of Prediction Models for Chemical Components in Alfalfa Blocks

To further examine the predictive behaviour of the optimal PLSR models, the measured and predicted values of the test set for CP, starch, fat, NDF, and ADF were compared (Figure 6). Because the modelling procedure was repeated over 20 random data partitions, Figure 6 presents the pooled test-set predictions from all repeats together with the ±1 RMSE band, while the corresponding test-set metrics in Table 2 are reported as mean ± standard deviation across the 20 repetitions. Overall, the pooled predictions followed the 1:1 reference line reasonably well, although the degree of agreement varied among constituents. This indicates generally consistent predictive behaviour within the present dataset and repeated-partition evaluation framework.

The starch model also showed satisfactory agreement between predicted and measured values, with a mean test-set R^2^ of 0.877 ± 0.023, an RMSE of 0.308 ± 0.022, and an RPD of 2.959 ± 0.291. For fat, the pooled predictions showed greater dispersion around the 1:1 line than those of CP and NDF, consistent with its comparatively lower predictive performance (R^2^ = 0.697 ± 0.058, RMSE = 0.181 ± 0.010, RPD = 1.883 ± 0.164). These results indicate that the FTIR-based PLSR model captured useful fat-related spectral information, although its quantitative predictive capability was more limited than that observed for CP, starch, and NDF.

For ADF, the model achieved a mean test-set R^2^ of 0.839 ± 0.031, an RMSE of 0.579 ± 0.041, and an RPD of 2.591 ± 0.287. The pooled predictions generally followed the 1:1 line, although a greater degree of scatter was observed than for NDF. This suggests that the combined preprocessing and CARS-based variable-selection strategy retained relevant spectral information for ADF prediction, while leaving greater residual variability than for NDF.

Overall, the pooled prediction plots support the quantitative results reported in Table 2. NDF and CP exhibited the strongest predictive agreement, followed by starch and ADF, whereas fat showed comparatively larger prediction uncertainty. Although no pronounced systematic departure from the 1:1 line was apparent in the pooled plots, these results should be interpreted within the present repeated-partition framework rather than as evidence of external generalisation.

## 4. Conclusions

This study demonstrated the feasibility of combining FTIR spectroscopy with machine-learning approaches to determine the chemical composition of alfalfa blocks. The application of multiple spectral preprocessing and feature-extraction methods enabled effective extraction of relevant FTIR information, facilitating rapid assessment of alfalfa block quality. Spectral preprocessing had a significant influence on the prediction performance of different chemical components. Among the evaluated approaches, prediction models developed using CARS feature selection combined with PLSR achieved the highest accuracy.

Different preprocessing methods may reduce spectral noise and background-related variation to different extents while emphasising different spectral features, thereby supporting the prediction of multiple chemical constituents. Baseline-related treatments had a substantial effect on the extracted spectral information and subsequent model performance. However, their effects were constituent-dependent and not uniformly beneficial. Although baseline correction can reduce offsets and drift associated with scattering and other background effects, incomplete correction may leave residual background variation, whereas overly aggressive correction may attenuate broad spectral features that contain chemically relevant information. Therefore, the instrument-based polynomial BC and airPLS were evaluated as alternative baseline-related procedures rather than being assumed to improve prediction universally; the most suitable preprocessing strategy was selected separately for each constituent according to validation performance.

The supervised CARS algorithm effectively reduced spectral dimensionality and improved prediction performance by selecting variables closely associated with the target chemical constituents. Overall, the developed prediction models provide a rapid, reagent-free laboratory method for estimating the five constituents from a single FTIR spectrum, with “rapid” indicating an analysis time considerably shorter than that of the reference wet-chemistry methods; this offers a practical tool for nutritional evaluation in livestock production. Nevertheless, the present study has a limitation in that complete batch traceability information was not available for all samples. Consequently, potential batch-related sources of variation could not be explicitly quantified or incorporated into the validation design, and the extent to which the models generalize across independent production batches remains to be further verified. Future studies should therefore include well-documented batch information and external validation using samples collected from multiple production batches, seasons, or sources. Against the backdrop of growing global feed trade and increasing demand for quality control, this study advances the application of FTIR spectroscopy in feed-quality analysis by integrating optimised preprocessing strategies and feature-extraction techniques, offering important theoretical and practical value.

## Figures and Tables

**Figure 1:**
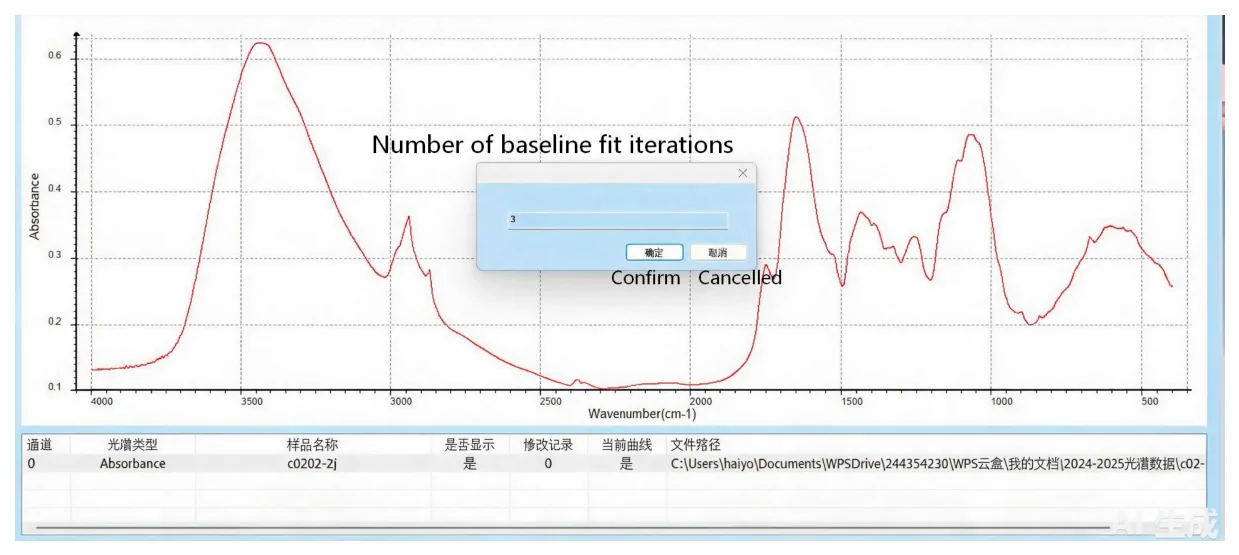
Fourier transform infrared spectrum baseline correction.

**Figure 2:**
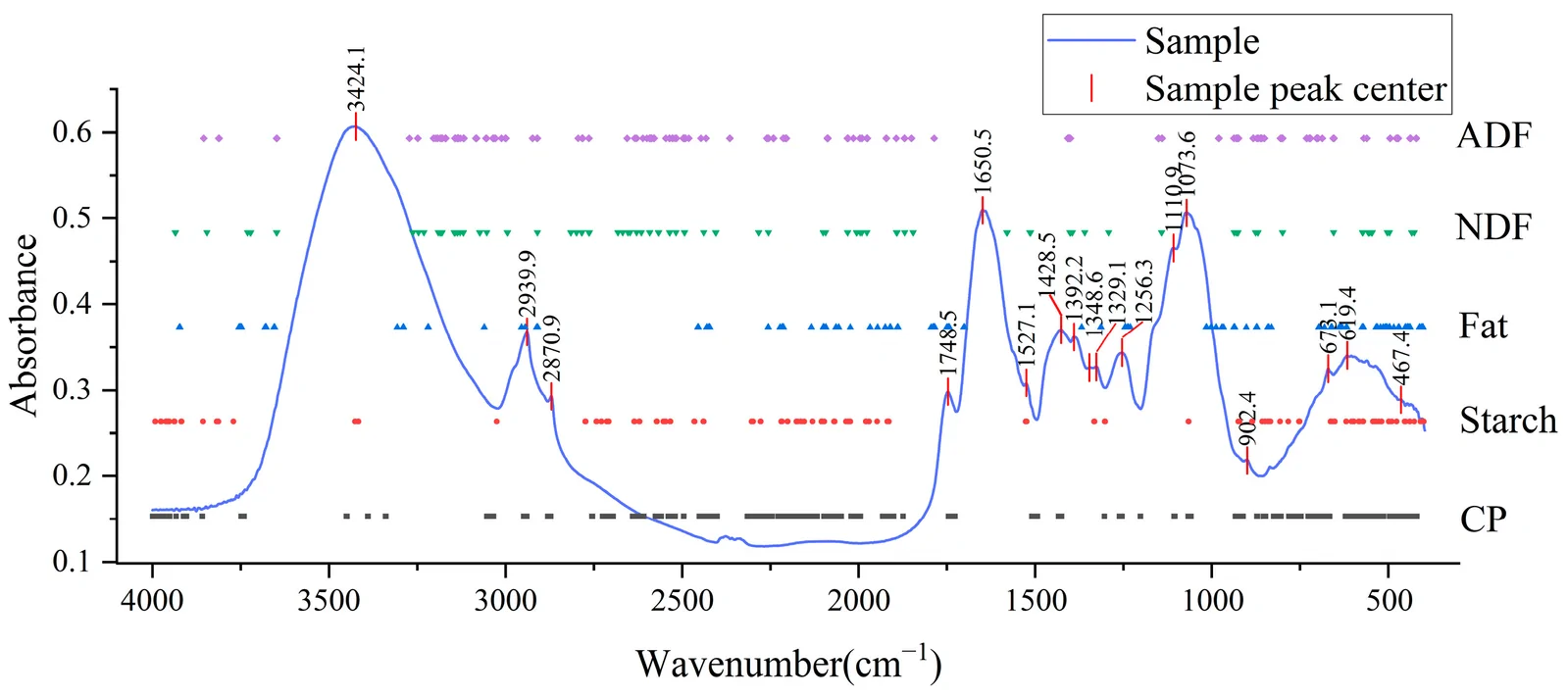
Distribution of CARS-selected spectral variables for CP, starch, fat, NDF, and ADF superimposed on the average FTIR spectrum of alfalfa blocks.

**Figure 3:**
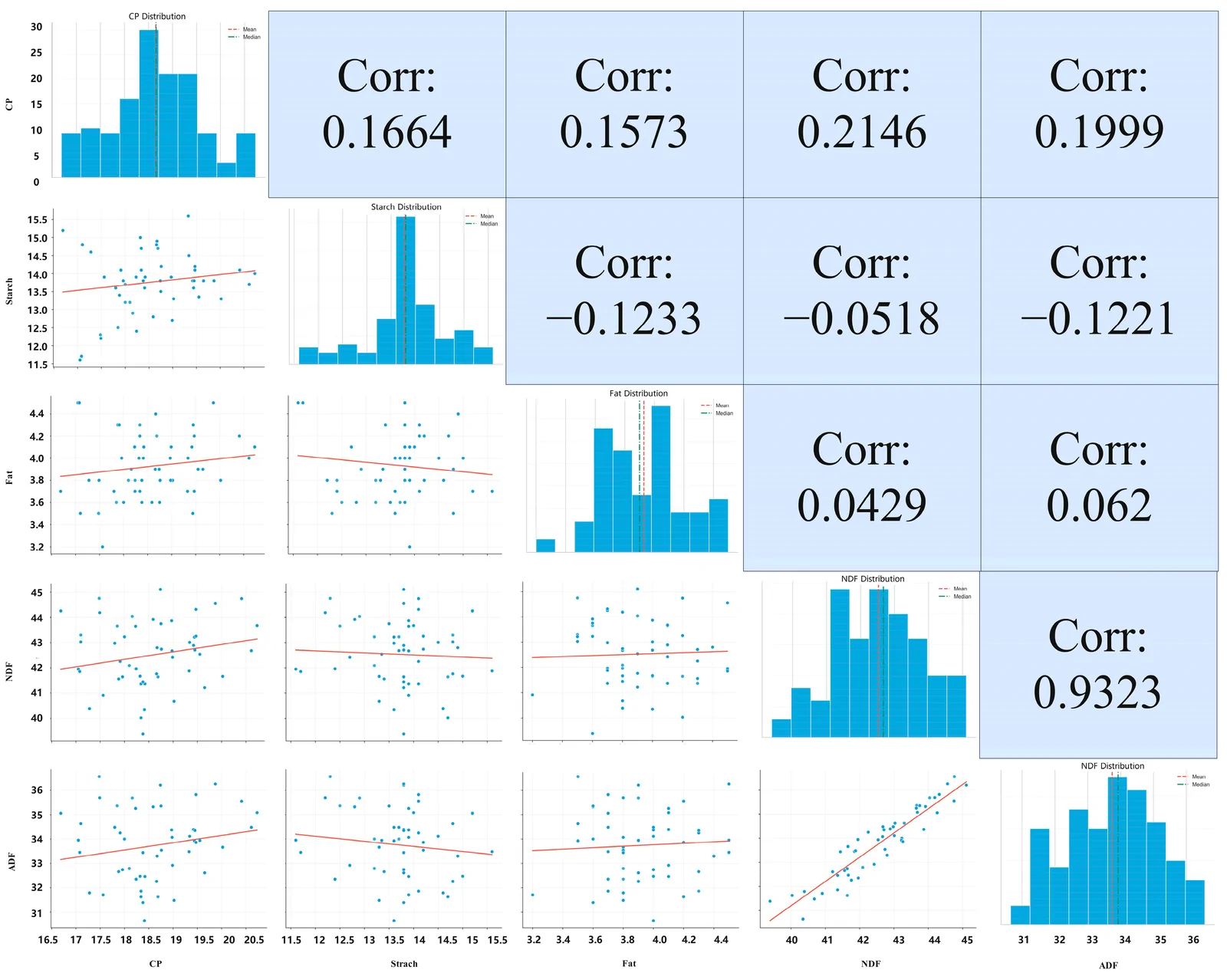
Scatter Plots of Correlations Among Chemical Composition Variables.

**Figure 4:**
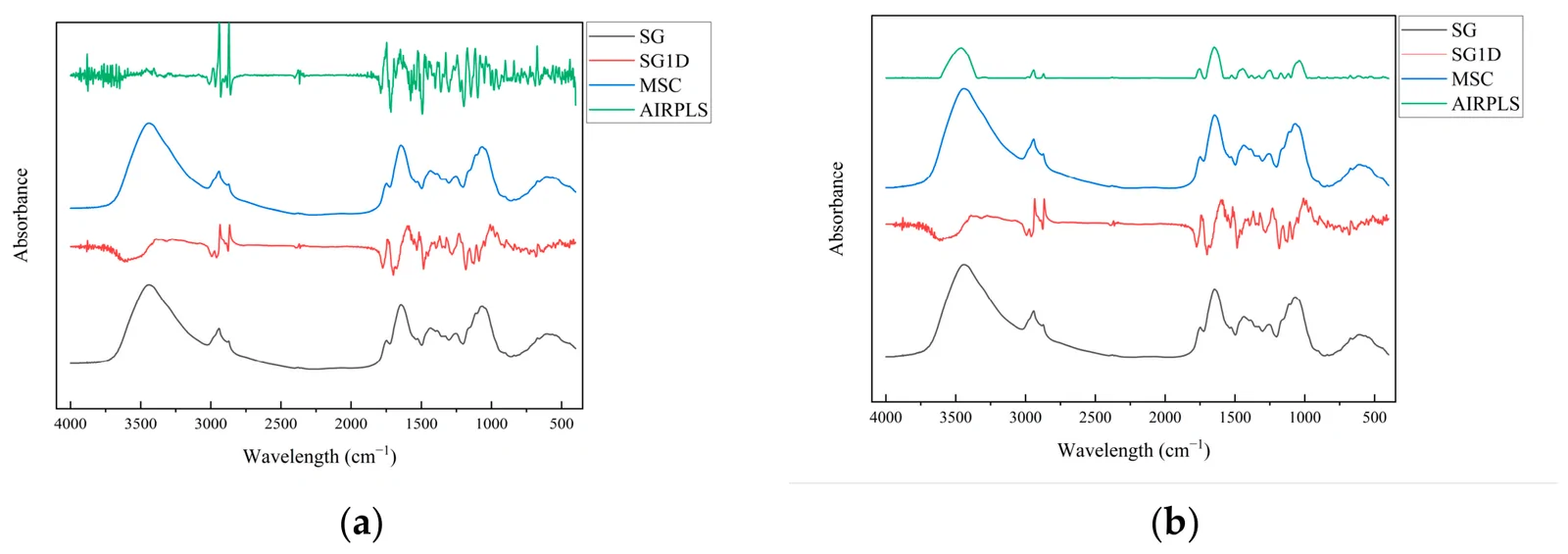
FTIR Spectral Preprocessing Results: (**a**) without baseline correction, (**b**) with baseline correction.

**Figure 5:**
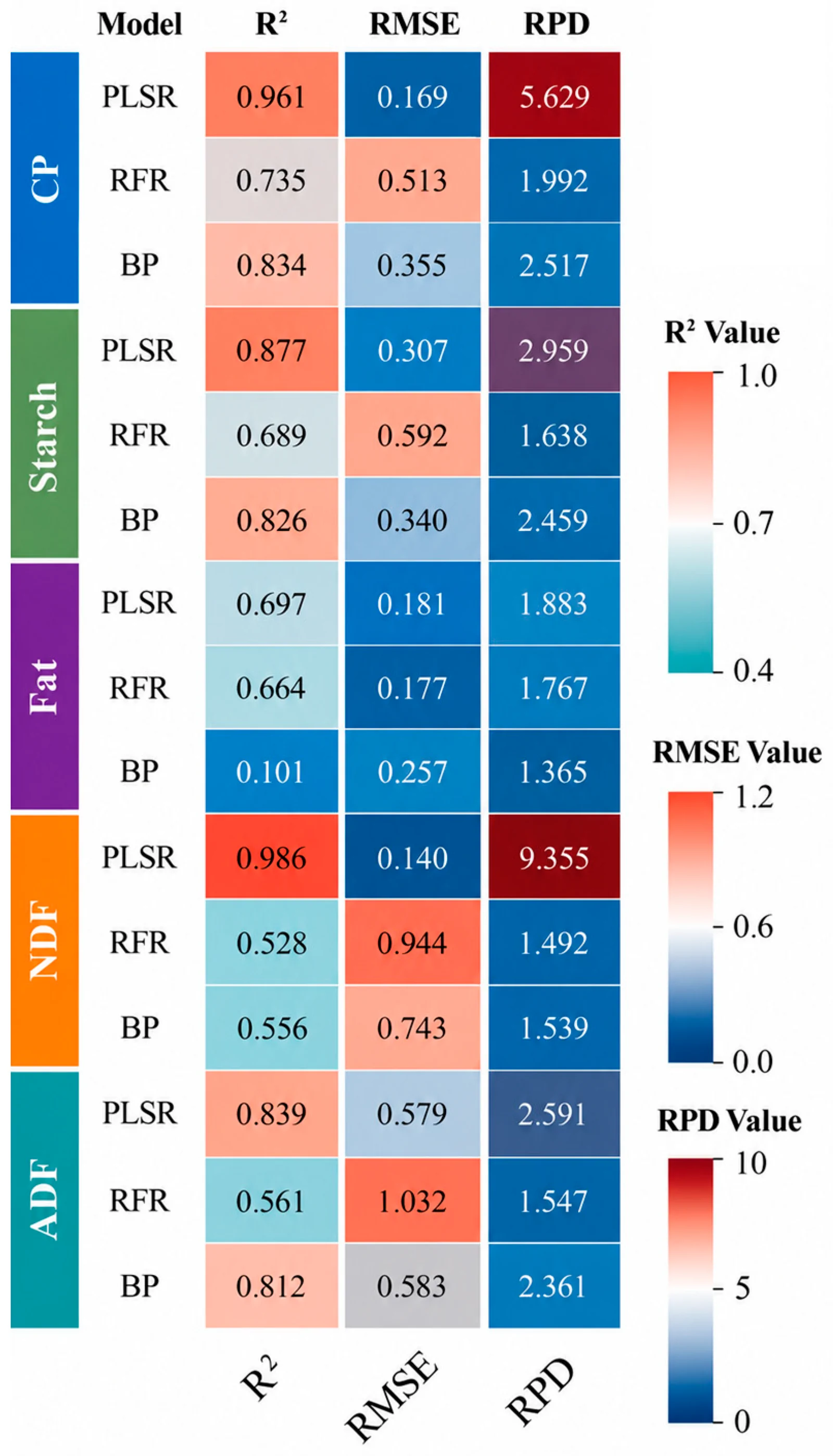
Comparison of R^2^, RMSE, and RPD among Three Regression Models Using Heatmaps. Note: Mean test-set R^2^, RMSE, and RPD values of PLSR, RFR, and BP models across 20 repeated random partitions. The same data partitions were used for the three model types to ensure direct comparability.

**Figure 6:**
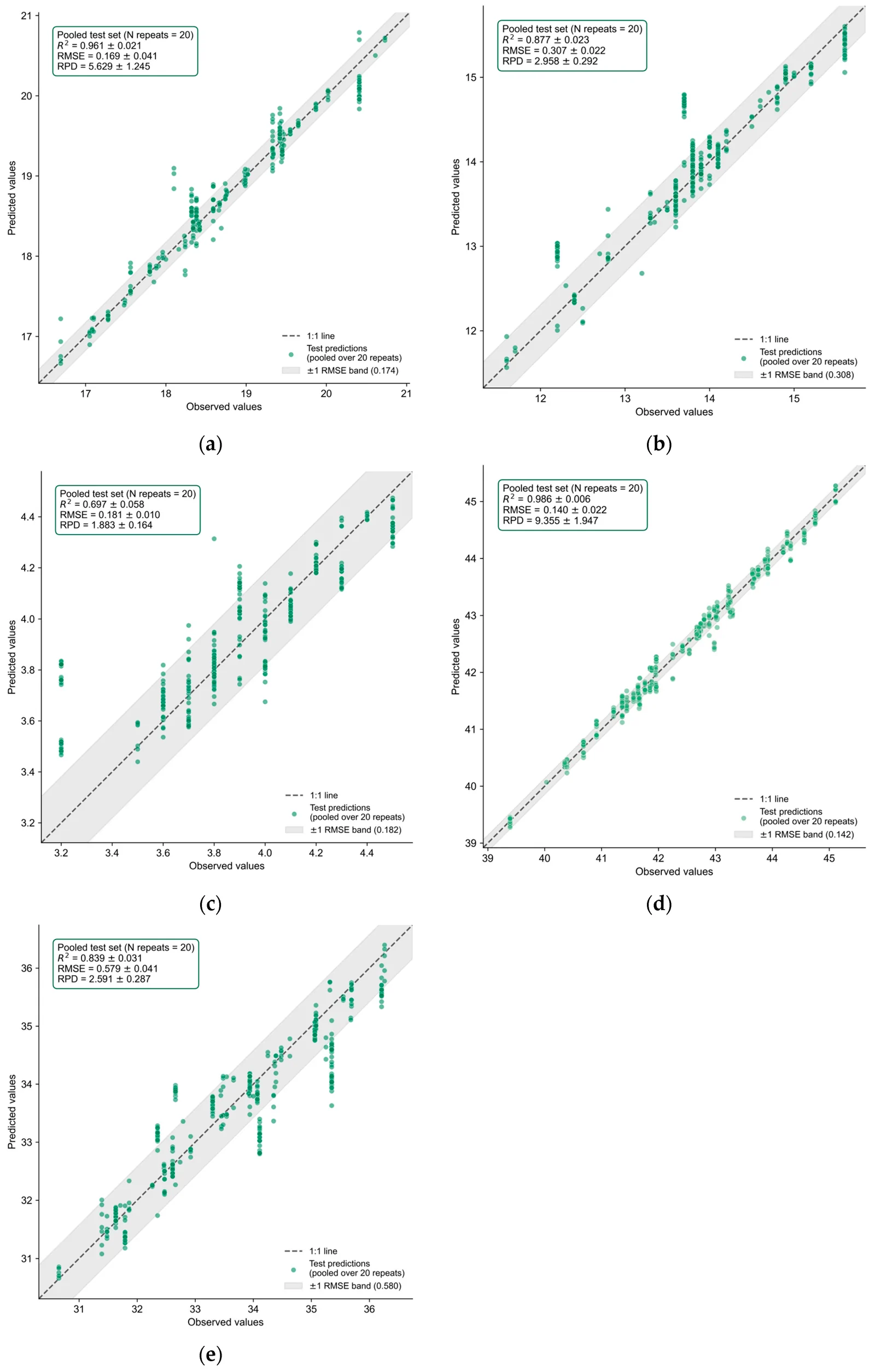
Prediction performance of chemical components in alfalfa blocks: (**a**) CP, (**b**) Starch, (**c**) Fat, (**d**) NDF, (**e**) ADF.

**Table 1:** Predictive Performance of PLSR Models Under Different Spectral Preprocessing Conditions.

Chemical Composition	Preprocessing	Pre-Order Preprocessing							
		None				BC			
		Training Set		Test Set		Training Set		Test Set	
		R^2^	RPD	R^2^	RPD	R^2^	RPD	R^2^	RPD
CP	SG	0.5234	1.4561	0.3404	1.2617	0.4272	1.3282	0.1978	1.144
	SG1D	0.998	22.311	0.9621	5.2627	0.5408	1.4836	0.443	1.373
	MSC	0.49	1.4078	0.3128	1.2361	0.4707	1.3818	0.2831	1.2102
	airPLS	0.2842	1.1883	0.2264	1.165	0.5159	1.4448	0.4609	1.3955
Fat	SG	0.0008	1.0057	−0.0157	1.0167	0.0162	1.0136	0.0119	1.0309
	SG1D	0.6782	1.7722	0.5235	1.4844	0.1481	1.0892	0.0725	1.064
	MSC	0.2692	1.176	0.1867	1.1362	0.8696	2.7841	0.5486	1.5252
	airPLS	0.6278	1.6478	0.2837	1.2107	0.7877	2.1821	0.4218	1.3476
Starch	SG	0.4679	1.3781	0.2565	1.1884	0.0895	1.0536	0.1932	1.1408
	SG1D	0.9943	13.3279	0.8898	3.0867	0.994	12.9802	0.8365	2.5343
	MSC	0.5535	1.5044	0.4442	1.3745	0.4219	1.3222	0.5132	1.4685
	airPLS	0.8179	2.3559	0.4974	1.4454	0.9914	10.8516	0.794	2.2579
NDF	SG	0.8006	2.2511	0.164	1.1207	0.5488	1.4966	−3.1173	0.505
	SG1D	0.6879	1.7994	0.2549	1.1871	0.9853	8.2783	−0.4573	0.8488
	MSC	0.7566	2.0377	0.4901	1.4351	0.7884	2.1855	0.3649	1.2858
	airPLS	0.7089	1.8633	0.0689	1.0619	0.981	7.2892	0.6278	1.6796
ADF	SG	0.6938	1.8169	0.3858	1.3075	0.1053	1.0628	0.2289	1.1669
	SG1D	0.6454	1.6881	0.245	1.1793	0.9906	10.3575	−0.5529	0.8223
	MSC	0.8011	2.2539	0.6334	1.6925	0.6536	1.7082	0.3565	1.2774
	airPLS	0.7145	1.8813	0.1472	1.1096	0.9656	5.4229	0.571	1.5644

**Table 2:** Comparison of PLSR Prediction Performance for Alfalfa Block Chemical Components Using Different Feature Extraction Methods.

Chemical Composition	Preprocessing	Feature Extraction	Training Set			Test Set		
			R^2^	RMSE	RPD	R^2^	RMSE	RPD
CP	SG1D	PCA	0.872 ± 0.014	0.337 ± 0.019	2.816 ± 0.156	0.696 ± 0.130	0.516 ± 0.097	1.955 ± 0.349
		SPA	0.882 ± 0.058	0.310 ± 0.091	3.569 ± 2.033	0.567 ± 0.182	0.614 ± 0.103	1.643 ± 0.316
		CARS	0.998 ± 0.001	0.039 ± 0.012	26.532 ± 7.212	0.961 ± 0.021	0.169 ± 0.041	5.629 ± 1.245
Starch	SG1D	PCA	0.733 ± 0.039	0.430 ± 0.027	1.962 ± 0.147	0.338 ± 0.274	0.618 ± 0.090	1.326 ± 0.236
		SPA	0.696 ± 0.199	0.428 ± 0.166	2.519 ± 1.717	0.129 ± 0.482	0.702 ± 0.120	1.173 ± 0.220
		CARS	0.995 ± 0.002	0.056 ± 0.012	15.369 ± 3.078	0.877 ± 0.023	0.308 ± 0.022	2.959 ± 0.291
Fat	BC-MSC	PCA	0.071 ± 0.023	0.278 ± 0.007	1.043 ± 0.013	−0.442 ± 0.975	0.344 ± 0.112	1.043 ± 0.013
		SPA	0.773 ± 0.156	0.131 ± 0.039	2.358 ± 0.558	0.126 ± 1.154	0.253 ± 0.150	1.398 ± 0.413
		CARS	0.957 ± 0.006	0.058 ± 0.003	4.895 ± 0.337	0.697 ± 0.058	0.181 ± 0.010	1.883 ± 0.164
NDF	BC-airPLS	PCA	0.890 ± 0.022	0.431 ± 0.042	3.077 ± 0.321	0.436 ± 0.285	0.948 ± 0.208	1.464 ± 0.314
		SPA	0.605 ± 0.104	0.813 ± 0.108	1.645 ± 0.243	−0.207 ± 0.794	1.376 ± 0.402	1.022 ± 0.207
		CARS	0.988 ± 0.002	0.140 ± 0.012	9.414 ± 0.870	0.986 ± 0.006	0.140 ± 0.022	9.355 ± 1.947
ADF	BC-airPLS	PCA	0.852 ± 0.026	0.540 ± 0.041	2.638 ± 0.214	0.468 ± 0.122	1.029 ± 0.148	1.425 ± 0.136
		SPA	0.515 ± 0.167	0.964 ± 0.181	1.531 ± 0.3689	−0.062 ± 0.446	1.440 ± 0.292	1.031 ± 0.139
		CARS	0.991 ± 0.003	0.131 ± 0.021	11.001 ± 1.714	0.839 ± 0.031	0.579 ± 0.041	2.591 ± 0.287

## Data Availability

The data presented in this study are available from the corresponding authors upon reasonable request. The dataset was generated through in-house testing and is not publicly available in order to protect privacy and confidentiality.

## Abbreviations

The following abbreviations are used in this manuscript:

FTIR	Fourier-transform infrared
CP	crude protein
NDF	neutral detergent fibre
ADF	acid detergent fibre
PLSR	partial least squares regression
RFR	random forest regression
BP	back-propagation
BC	baseline correction
CARS	competitive adaptive reweighted sampling
SG1D	first-derivative Savitzky–Golay preprocessing
SG	Savitzky–Golay preprocessing
MSC	multiplicative scatter correction
airPLS	adaptive iteratively reweighted penalised least squares
SPA	successive projections algorithm
PCA	principal component analysis
RPD	residual predictive deviation
RMSE	root mean square error
R^2^	coefficient of determination

## References

[1] Golea C.M., Codină G.G., Oroian M. (2023). Prediction of wheat flours composition using fourier transform infrared spectrometry (FT-IR). Food Control.

[2] Refat B., Yu P. (2022). Evaluation of prediction of indigestible fiber fraction (iNDF) of whole-crop barley silage by using non-destructive spectroscopic techniques as a fast-screening method: Comparison between FTIR vs. NIR. Can. J. Plant Sci..

[3] Lohumi S., Lee S., Lee W., Kim M.S., Mo C., Bae H., Cho B. (2014). Detection of Starch Adulteration in Onion Powder by FT-NIR and FT-IR Spectroscopy. J. Agric. Food. Chem..

[4] Du H., Lei T., Ma Y., Li Y., Yang X., Du X. (2024). Effect of Process Parameters on Quality of Alfalfa Block. Bioresources.

[5] Du H., Lei T., Ma Y., Su H., Xuan C. (2024). Influence and prediction of chemical composition of alfalfa on the quality and specific energy consumption of alfalfa briquettes. Ind. Crop. Prod..

[6] Palmonari A., Fustini M., Canestrari G., Grilli E., Formigoni A. (2014). Influence of maturity on alfalfa hay nutritional fractions and indigestible fiber content. J. Dairy Sci..

[7] Zheng C., Liu H., Li J., Wang Y. (2024). Identification and crude protein prediction of porcini mushrooms via deep learning-assisted FTIR fingerprinting. LWT.

[8] Niemi C., Mortensen A.M., Rautenberger R., Matsson S., Gorzsás A., Gentili F.G. (2023). Rapid and accurate determination of protein content in North Atlantic seaweed by NIR and FTIR spectroscopies. Food Chem..

[9] Wu M., Li Y., Yuan Y., Li S., Song X., Yin J. (2023). Comparison of NIR and Raman spectra combined with chemometrics for the classification and quantification of mung beans (*Vigna radiata* L.) of different origins. Food Control.

[10] Shi S., Zhao D., Pan K., Ma Y., Zhang G., Li L., Cao C., Jiang Y. (2023). Combination of near-infrared spectroscopy and key wavelength-based screening algorithm for rapid determination of rice protein content. J. Food Compos. Anal..

[11] Du H., Zhang Y., Ma Y., Jiao W., Lei T., Su H. (2024). Rapid Determination of Crude Protein Content in Alfalfa Based on Fourier Transform Infrared Spectroscopy. Foods.

[12] Liu J., Luo X., Zhang D., Wang C., Chen Z., Zhao X. (2023). Rapid determination of rice protein content using near-infrared spectroscopy coupled with feature wavelength selection. Infrared Phys. Technol..

[13] Liu W., Zhou Q., Yang S., Song F., Li Z., Wang J., Song C., Ling C. (2026). Quality grading of Zijin cicada tea using a multi-level fusion strategy. Food Control.

[14] Fei X., Jiang X., Lei Y., Tian J., Hu X., Bu Y., Huang D., Luo H. (2023). The Rapid Non-Destructive Detection of the Protein and Fat Contents of Sorghum Based on Hyperspectral Imaging. Food Anal. Methods.

[15] Huang J., Zeng L., Wei S., Tong H., Ji X., Wu M., Yang Y. (2024). NIRS prediction of the protein, fat, and ash of Sargassum fusiforme at different growth stages: A quality control approach. Infrared Phys. Technol..

[16] Luan M., Wang H., Wang S., Zhou J., Wan X., Hong J., Yang J., Bai R. (2025). Hyperspectral imaging combined with chemometrics for rapid detection of Poria cocos adulteration: A qualitative and quantitative approach. J. Food Compos. Anal..

[17] (2018). Determination of Crude Protein in Feed—Kjeldahl Method.

[18] (2006). Determination of Crude Fat in Feed.

[19] (2022). Determination of Neutral Detergent Fibre in Feed.

[20] (2022). Determination of Acid Detergent Fibre in Feed.

[21] (2023). Determination of Starch in Foods.

[22] Lazzari E., Schena T., Marcelo M.C.A., Primaz C.T., Silva A.N., Ferrão M.F., Bjerk T., Caramão E.B. (2018). Classification of biomass through their pyrolytic bio-oil composition using FTIR and PCA analysis. Ind. Crop. Prod..

[23] Tang R., Chen X., Li C. (2018). Detection of Nitrogen Content in Rubber Leaves Using Near-Infrared (NIR) Spectroscopy with Correlation-Based Successive Projections Algorithm (SPA). Appl. Spectrosc..

[24] Widyaningrum, Purwanto Y.A., Widodo S., Supijatno, Iriani E.S. (2024). Rapid assessment of vanilla (*Vanilla planifolia*) quality parameters using portable near-infrared spectroscopy combined with random forest. J. Food Compos. Anal..

[25] Kaur S., Singh N., Dagar P., Kumar A., Jaiswal S., Singh B.K., Bhardwaj R., Chand Rana J., Riar A. (2024). Comparative analysis of modified partial least squares regression and hybrid deep learning models for predicting protein content in Perilla (*Perilla frutescens* L.) seed meal using NIR spectroscopy. Food Biosci..

[26] Hssaini L., Razouk R., Bouslihim Y. (2022). Rapid Prediction of Fig Phenolic Acids and Flavonoids Using Mid-Infrared Spectroscopy Combined with Partial Least Square Regression. Front. Plant Sci..

[27] Schütz D., Riedl J., Achten E., Fischer M. (2022). Fourier-transform near-infrared spectroscopy as a fast screening tool for the verification of the geographical origin of grain maize (*Zea mays* L.). Food Control.

[28] Christou C., Agapiou A., Kokkinofta R. (2018). Use of FTIR spectroscopy and chemometrics for the classification of carobs origin. J. Adv. Res..

[29] Lewis R.N.A.H., McElhaney R.N. (2007). Fourier Transform Infrared Spectroscopy in the Study of Lipid Phase Transitions in Model and Biological Membranes. Lewis Mcelhaney.

[30] Orozco R.S., Hernández P.B., Morales G.R., Núñez F.U., Villafuerte J.O., Lugo V.L., Ramírez N.F., Díaz C.E.B., Vázquez P.C. (2014). Characterization of Lignocellulosic Fruit Waste as an Alternative Feedstock for Bioethanol Production. Bioresources.

[31] Huang H., Wu D., Fang L., Zheng X. (2022). Comparison of Multiple Machine Learning Models for Estimating the Forest Growing Stock in Large-Scale Forests Using Multi-Source Data. Forests.

[32] Gong Y., Chen X., Wu W. (2024). Application of fourier transform infrared (FTIR) spectroscopy in sample preparation: Material characterization and mechanism investigation. Adv. Sample Prep..

[33] Javier-Astete R., Jimenez-Davalos J., Zolla G. (2021). Determination of hemicellulose, cellulose, holocellulose and lignin content using FTIR in *Calycophyllum spruceanum* (Benth.) K. Schum. and *Guazuma crinita* Lam. PLoS ONE.

[34] Lahlali R., Jiang Y., Kumar S., Karunakaran C., Liu X., Borondics F., Hallin E., Bueckert R. (2014). ATR-FTIR spectroscopy reveals involvement of lipids and proteins of intact pea pollen grains to heat stress tolerance. Front. Plant Sci..

[35] Meng X., Bao Y., Liu J., Liu H., Zhang X., Zhang Y., Wang P., Tang H., Kong F. (2020). Regional soil organic carbon prediction model based on a discrete wavelet analysis of hyperspectral satellite data. Int. J. Appl. Earth Obs. Geoinf..

[36] Sun X., Li J., Shen Y., Li W. (2021). Non-destructive Detection of Insect Foreign Bodies in Finishing Tea Product Based on Terahertz Spectrum and Image. Front. Nutr..

[37] Gvaladze S., De Roover K., Tuerlinckx F., Ceulemans E. (2021). Detecting outlying variables in multigroup data: A comparison of different loading similarity coefficients. J. Chemom..

[38] Wu X., Zeng S., Fu H., Wu B., Zhou H., Dai C. (2023). Determination of corn protein content using near-infrared spectroscopy combined with A-CARS-PLS. Food Chem. X.

